# Decay-Accelerating Factor 1 Deficiency Exacerbates Leptospiral-Induced Murine Chronic Nephritis and Renal Fibrosis

**DOI:** 10.1371/journal.pone.0102860

**Published:** 2014-07-17

**Authors:** María F. Ferrer, Emilia Scharrig, Lucrecia Alberdi, Maia Cedola, Gabriela Pretre, Ricardo Drut, Wen-Chao Song, Ricardo M. Gomez

**Affiliations:** 1 Instituto de Biotecnología y Biología Molecular, CONICET-UNLP, La Plata, Buenos Aires, Argentina; 2 Cátedra de Patología “A”, Facultad de Medicina, UNLP, La Plata, Buenos Aires, Argentina; 3 Department of Pharmacology, Perelman School of Medicine, University of Pennsylvania, Philadelphia, Pennsylvania, United States of America; Cornell University, United States of America

## Abstract

Leptospirosis is a global zoonosis caused by pathogenic *Leptospira*, which can colonize the proximal renal tubules and persist for long periods in the kidneys of infected hosts. Here, we characterized the infection of C57BL/6J wild-type and Daf1^−/−^ mice, which have an enhanced host response, with a virulent *Leptospira interrogans* strain at 14 days post-infection, its persistence in the kidney, and its link to kidney fibrosis at 90 days post-infection. We found that *Leptospira interrogans* can induce acute moderate nephritis in wild-type mice and is able to persist in some animals, inducing fibrosis in the absence of mortality. In contrast, Daf1^−/−^ mice showed acute mortality, with a higher bacterial burden. At the chronic stage, Daf1^−/−^ mice showed greater inflammation and fibrosis than at 14 days post-infection and higher levels at all times than the wild-type counterpart. Compared with uninfected mice, infected wild-type mice showed higher levels of IL-4, IL-10 and IL-13, with similar levels of α-smooth muscle actin, galectin-3, TGF-β1, IL-17, IFN-γ, and lower IL-12 levels at 90 days post-infection. In contrast, fibrosis in Daf1^−/−^ mice was accompanied by high expression of α-smooth muscle actin, galectin-3, IL-10, IL-13, and IFN-γ, similar levels of TGF-β1, IL-12, and IL-17 and lower IL-4 levels. This study demonstrates the link between *Leptospira*-induced murine chronic nephritis with renal fibrosis and shows a protective role of Daf1.

## Introduction

Renal fibrosis is a reliable predictor of prognosis, a major determinant of renal insufficiency and a common final outcome of almost all progressive chronic kidney diseases (CKD) [1], [2]. In USA the prevalence of CKD is increasing and affects an estimated 13% of the population [3] or even more in developing countries [4]. The etiology of CKD in developed countries is associated with atherosclerosis, diabetes, and autoimmune glomerulonephritis [2]. However, it is possible that in developing countries infections have also a significant impact.

Leptospirosis is a global zoonosis caused by spirochetes of the genus *Leptospira* [5]. Human infection commonly occurs through direct contact with infected animal urine or indirectly through contaminated water. Almost every mammal can serve as a carrier of leptospires, harboring the spirochete in the proximal renal tubules of the kidneys, leading to urinary shedding. Rodents, mainly rats, serve as the major carriers in most human leptospirosis, excreting high concentrations of leptospires (10^7^ organisms per ml) months after their initial infections [6]. Consequently, leptospirosis can be considered a disease with an endemo-epidemic pattern associated with slum settlements [7]. Leptospirosis is usually a biphasic disease with an early bacteremic phase during which leptospires are disseminated rapidly throughout the body during the first 7–10 days of infection. This is followed by a leptospiruric phase during which specific antibodies arise in parallel with the disappearance of the bacteria from the blood and most organs with the exception of the kidneys, where it can persist for long periods of time [8]. Although it can cause death or very severe symptoms (Weil’s syndrome), most documented cases are mild and self-limiting [9]. It is accepted that an accurate diagnosis of leptospirosis is frequently lacking.

In experimental leptospirosis, animal species and strain, age, dose inoculum, bacterial history, passage and *Leptospira* serovar influence the infection outcome [9], [10]. Guinea pigs and hamsters are the most commonly used animal experimental models for studying acute parameters [11], [12], whereas rats are considered a suitable model for studying persistent renal colonization [13], [14]. In contrast, mice are relatively more resistant, presenting fewer symptoms and lesions and lower mortality, and are therefore used less as a model [10]. However, mice may become more susceptible via immunosuppression [15], or by deletion of specific cytokine genes [16], suggesting that the immune response plays a major role in murine infection.

Decay-accelerating factor 1 (Daf1 or CD55) is a glycosylphosphatidylinositol-anchored membrane protein of the complement-regulatory family that protects cells from autologous complement attack [17]. Daf1 inhibits the assembly and accelerates the rapid decay of C3 and C5 convertases in both the classical and alternative complement activation pathways [17]. Interestingly, many kidney pathologies have been linked to abnormal complement activation [18]. It has been reported that Daf1^−/−^ mice are more susceptible to complement-mediated inflammatory injury and have significantly enhanced T-cell responses to active immunization. This phenotype is characterized by hypersecretion of interferon (IFN)-γ and IL-2 as well as down-regulation of the inhibitory cytokine IL-10 during antigen restimulation of lymphocytes *in vitro* [19]. Furthermore, several studies have shown that the absence of Daf1 exacerbates disease in a variety of autoimmune models, including systemic autoimmune disease in the MLR-Faslpr mouse [20], [21], focal and segmental glomerulosclerosis [22] and mercury-induced autoimmunity [23], by enhancing T-cell and autoimmune responses associated with hypersecretion of IFN-γ, IL-12 and IL-17 [19], [24], [25], [26].

Galectin-3 (Gal-3) is a β-galactoside-binding animal lectin [27]. Gal-3 expression and secretion by macrophages is a major mechanism linking macrophages to fibroblast activation and myofibroblast accumulation, as demonstrated by their synthesis of α-smooth muscle actin (α-SMA), thus driving renal fibrosis in the unilateral ureteric obstruction model (UUO) [28].

We hypothesized that by using a reference strain with known virulence, mice may become susceptible to chronic experimental leptospirosis. If successful, we could then explore whether the persistent renal infection affects the renal extracellular matrix (ECM) and explore some of the mechanisms involved, particularly the influence of an enhanced host response. In the present paper, we report the establishment of a murine model of chronic leptospirosis followed by fibrosis in wild-type and Daf1^−/−^ mice.

## Results

### *Leptospira interrogans* induces discrete to moderate nephritis in mice

In order to investigate whether *Leptospira interrogans,* serovar Copenhageni (LIC), was able to induce murine renal inflammation, and to study how an enhanced host response could impact in the disease, C57BL/6J wild-type (WT) and Daf1^−/−^ mice of 3–4 weeks of age were infected with 10^6^ bacteria and groups of five animals were euthanized and necropsied at 14 and 90 days post-infection (dpi).

**Figure 1:**
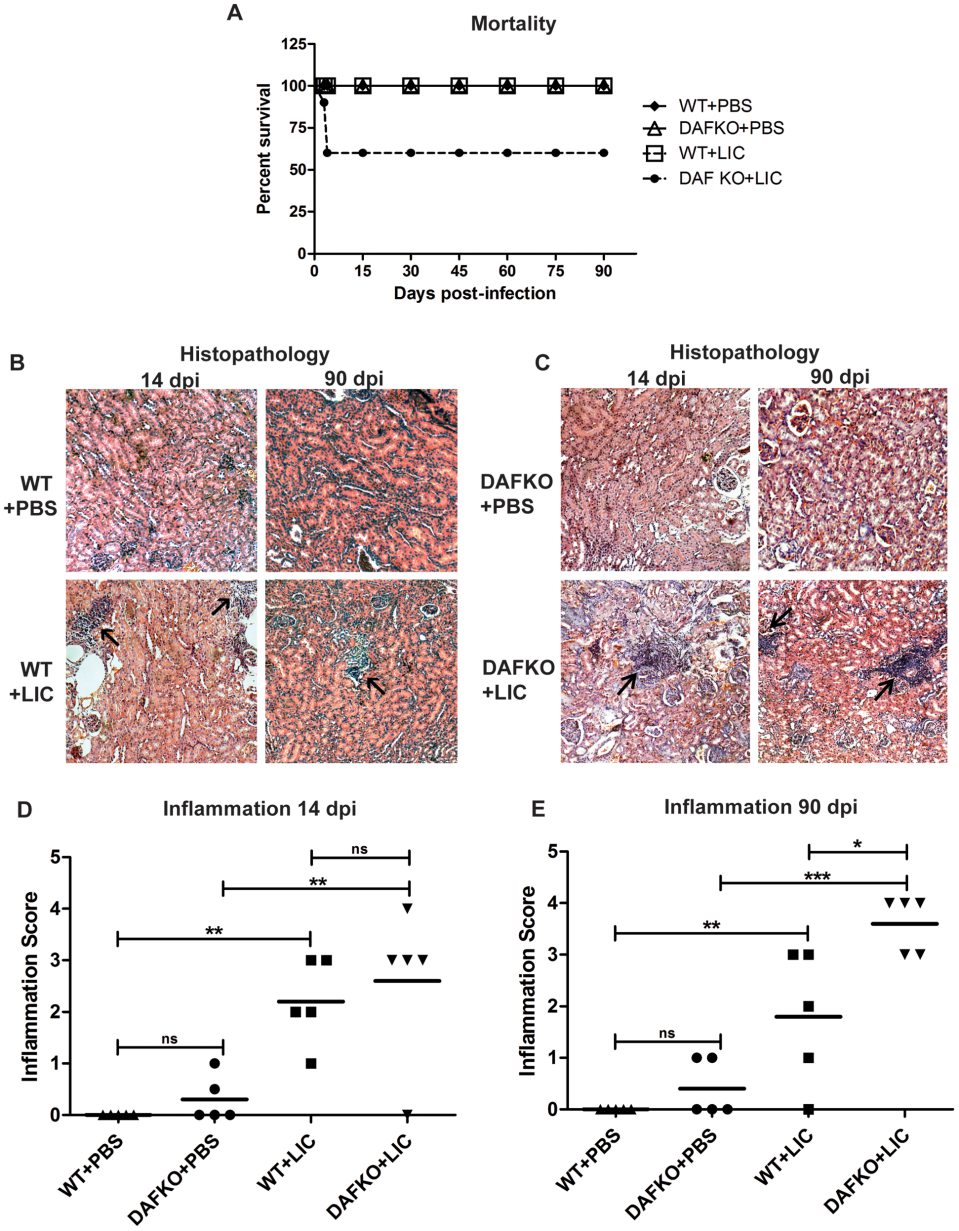
Leptospiral-induced nephritis in C57BL/6J wild-type and Daf1^−/−^ mice at 14 and 90 dpi. **A)** Survival percentage of WT+PBS (dark rhombus), WT+LIC (white square), DAFKO+PBS (white triangle) and DAFKO+LIC (dark circle) mice. Mice were monitored daily and euthanized by CO_2_ overdose at 90 dpi (5–7 mice were used). Mortality of C57BL/6J Daf1^−/−^ LIC-infected mice at 3 or 4 dpi was unexpected and presented no previous signs. **B–C)** Histopathologic sections of uninfected (WT+PBS and DAFKO+PBS) and infected C57BL/6J wild-type (WT+LIC) and Daf1^−/−^ (DAFKO+LIC) kidneys at 14 and 90 dpi assessed by hematoxylin and eosin staining (x200). Lymphomonocytic-rich infiltrates are indicated by arrows. **D–E)** Inflammation score of uninfected and LIC-infected C57BL/6J wild-type and Daf1^−/−^ animals. Mean inflammation score is represented by a straight line; *p<0.05, **p<0.01, ***p<0.001, **^ns^** *p*>0.05.

None of the uninfected (WT+PBS and Daf1^−/−^+PBS) or WT+LIC mice died during the experiment. In contrast, the Daf1^−/−^+LIC mice had 40% mortality at 4 dpi (Figure 1 A). No pathology was observed in uninfected mice (Figure 1 B–C). In contrast, infected animals developed interstitial nephritis in the form of multifocal lymphomonocytic infiltrates (Figure 1 B–C). The degree of inflammation was slightly higher (although not significant) in the Daf1^−/−^+LIC than in WT littermates at 14 dpi, but significantly higher at 90 dpi (*p*<0.05, Figure 1 D–E), when only 80% of WT+LIC mice showed inflammation. Taken together, these results show that Daf1^−/−^+LIC mice have greater susceptibility to infection than their WT littermates.

### Bacterial burden is significantly reduced at later time points

**Figure 2:**
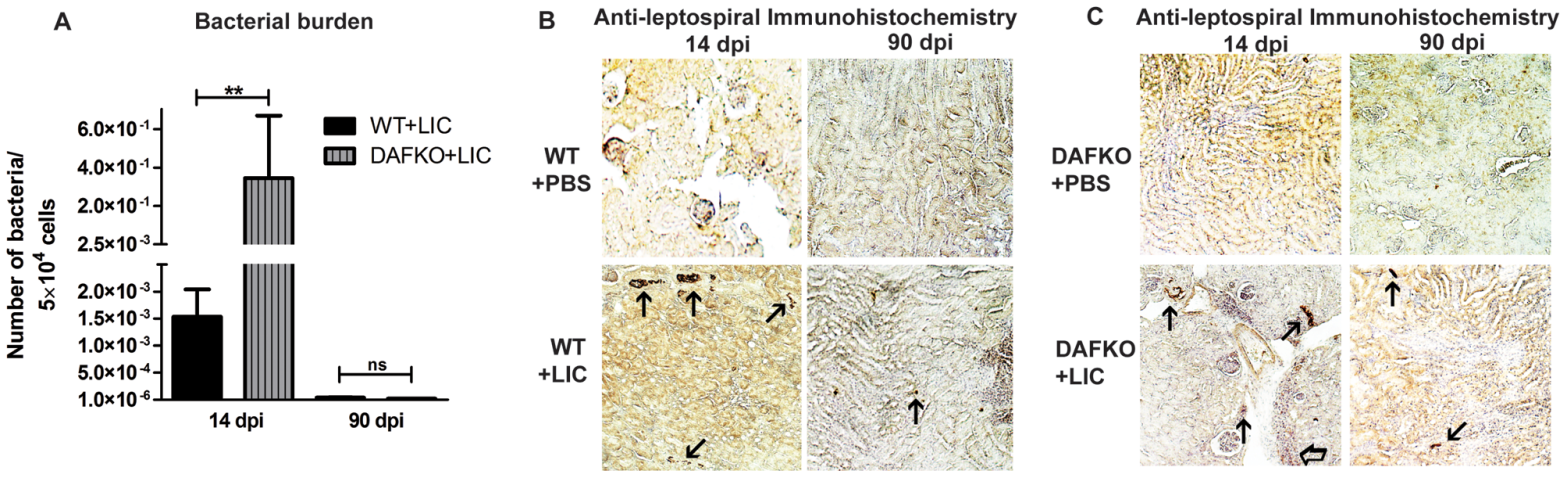
Bacterial burden is significantly reduced at later time points. **A)** Quantitative measurement of leptospiral DNA (16S) in kidney samples from WT (WT+LIC) or Daf1^−/−^ (DAFKO+LIC) infected animals with 10^6^ bacteria at 14 and 90 dpi. Bars represent standard error mean (SEM) of assays from a group of five to seven mice. Three pieces of each organ were analyzed in triplicate q-PCR and normalized to host cell number; **p<0.01, **^ns^** *p*>0.05. **B–C)** Immunohistochemistry with antiserum specific for LipL32 (x200) of kidney sections of uninfected (WT+PBS and DAFKO+PBS) and infected WT (WT+LIC) and Daf1^−/−^ (DAFKO+LIC) animals at 14 and 90 dpi. Arrows indicate representative positive foci.

Kidney bacterial burden showed an unexpected 200-fold increase (3.48×10^−1^ bacteria/5×10^4^ cells) in Daf1^−/−^ infected mice compared with WT mice at 14 dpi (1.53×10^−3^ bacteria/5×10^4^ cells, *p*<0.01, Figure 2 A). In contrast, the kidney bacterial burden was markedly reduced in both Daf1^−/−^ mice (1.4×10^−5^bacteria/5×10^4^ cells) and WT infected animals (3.9×10^−5^ bacteria/5×10^4^ cells) at 90 dpi (Figure 2 A). Immunohistochemistry (IHC) showed that the tissue distribution of leptospiral antigen was present mostly on the luminal surface of the proximal tubules (Figure 2 B–C), and occasionally inside macrophages and in the interstitium. A moderate number of antigen spots were observed in samples of mice harvested at 14 dpi, but leptospiral antigen became almost undetectable in samples from both groups of mice at 90 dpi (Figure 2 B–C).

### LIC-induced chronic nephritis produces renal fibrosis

In order to investigate whether the chronic nephritis induced in C57BL/6J mice infected with LIC was linked to fibrosis, renal collagen deposition was observed with Masson’s trichrome and digitally analyzed using Picro sirius red (PS) staining.

**Figure 3:**
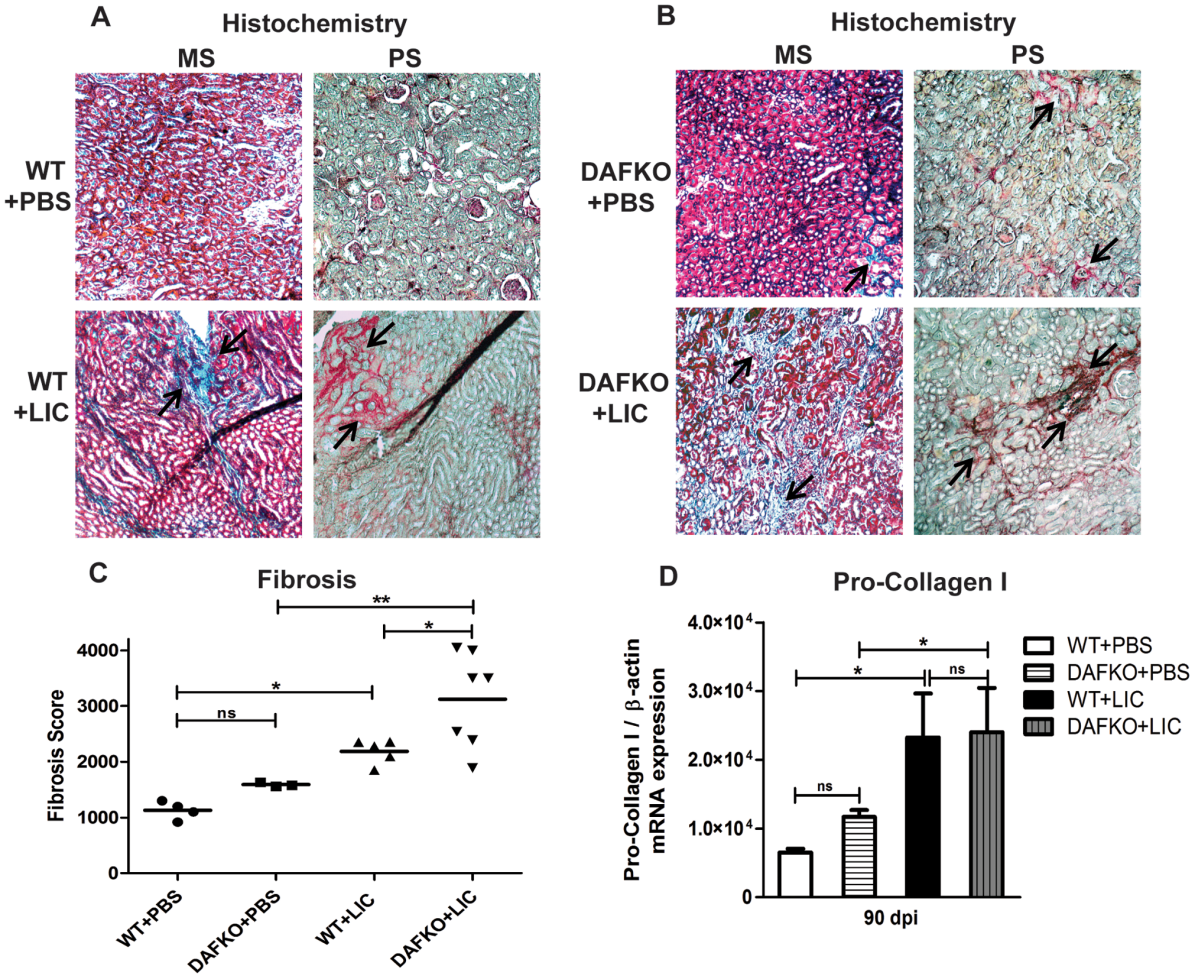
LIC-induced chronic nephritis produces renal fibrosis. **A–B)** Renal collagen deposition (indicated by arrows) was analyzed by Masson’s trichrome (MS) and Picro sirius red (PS) staining in uninfected (WT+PBS and DAFKO+PBS) and infected (WT+LIC and DAFKO+LIC) animals at 90 dpi (x200). **C)** Fibrosis score of WT (WT+PBS and WT+LIC) and Daf1^−/−^ (DAFKO+PBS and DAFKO+LIC) animals. Mean fibrosis score is represented by a straight line, *p<0.05, **p<0.01, **^ns^** *p>*0.05. **D)** Quantitative measurement of pro-collagen I mRNA in kidney samples from uninfected or infected animals at 90 dpi. Bars represent the standard error of the mean (SEM) of assays from a group of five mice. Three pieces of each organ were analyzed in triplicate for q-PCR and normalized to host β-actin expression; *p<0.05, **^ns^** *p>*0.05.

Uninfected animals had no signs of fibrosis during the experiment (Figure 3 A–B). On the other hand, renal interstitial fibrosis was evident with increased numbers of red collagen fibers, frequently observed away from lymphocyte-rich infiltrates in WT+LIC animals and even more in Daf1^−/−^ infected mice (*p*<0.05 and *p*<0.01, respectively, Figure 3 A–C), involving all animals in correlation with the higher inflammation observed but in contrast to the low *Leptospira* presence at 90 dpi.

The levels of pro-collagen I mRNA were similarly increased in both groups of infected mice compared with uninfected animals at 90 dpi (Figure 3 D, *p*<0.05).

### *Leptospira interrogans-*induced humoral response

**Figure 4:**
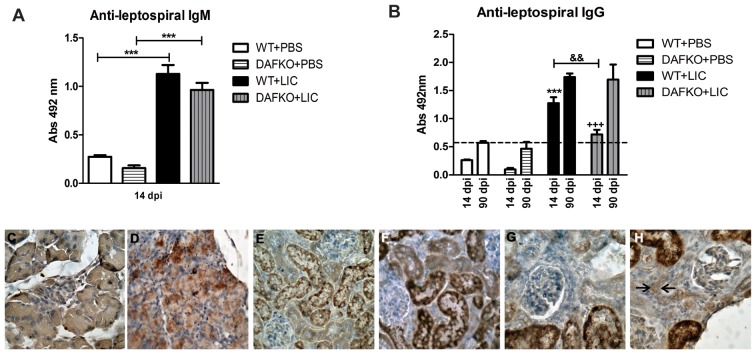
Anti-leptospiral antibodies followed by enhanced complement activation in Daf1^−/−^ mice suggest a minimal contribution to kidney damage. **A)** Total anti-leptospiral IgM from serum samples of uninfected and infected animals at 14 dpi was analyzed by ELISA. Bars represent standard error mean (SEM) of assays from a group of five to seven mice; ***p<0.001. **B)** Total anti-leptospiral IgG from serum samples of uninfected and infected animals at 14 and 90 dpi was analyzed by ELISA. Bars represent the SEM of assays from a group of five to seven mice; ***p<0.001 with respect to WT+PBS at 14 dpi; ^+++^ *p*<0.001 with respect to DAFKO+PBS at 14 dpi; ^&&^ *p*<0.01 between both WT and Daf1^−/−^ LIC-infected groups. Values under the baseline (dashed line) are considered negligible. Immunohistochemistry with antiserum specific for MAC (membrane attack complex) at 90 dpi (×200). **C)** Uninfected pancreas as the negative control, **D)** coxsackievirus B3-infected pancreas as the positive control, WT+LIC without (**E**) or with anti-MAC (**F**), DAFKO+LIC without (**G**) or with anti-MAC (**H**). Only in panel H there is minimal, positive labeling in interstitial cells indicated by arrows. Samples were treated according to Abcam’s suggested protocol including unmasking. Unmasking usually strongly increases the staining of kidney acinar cells which are rich in endogenous peroxidase, but this staining is intracytoplasmic.

Anti-leptospiral IgM levels in serum samples of Daf1^−/−^ infected mice at 14 dpi were higher than in uninfected mice (Figure 4 A, *p*<0.001), but similar to WT mice. In contrast, the IgG-specific humoral immune response was higher in WT than in the Daf1^−/−^ infected group at 14 dpi, but reached similarly high values in the infected groups at 90 dpi (Figure 4 B, *p*<0.01). In order to study if anti-leptospiral antibody production and enhanced complement activation contributed to kidney damage, we analyzed the membrane attack complex (MAC) tissue distribution by IHC and observed minimal MAC presence in Daf1^−/−^ infected mice at 90 dpi (Figure 4 C–H).

### Chronic fibrosis in Daf1^−/−^ LIC-infected mice correlates with myofibroblast activation and enhanced Gal-3 expression

**Figure 5:**
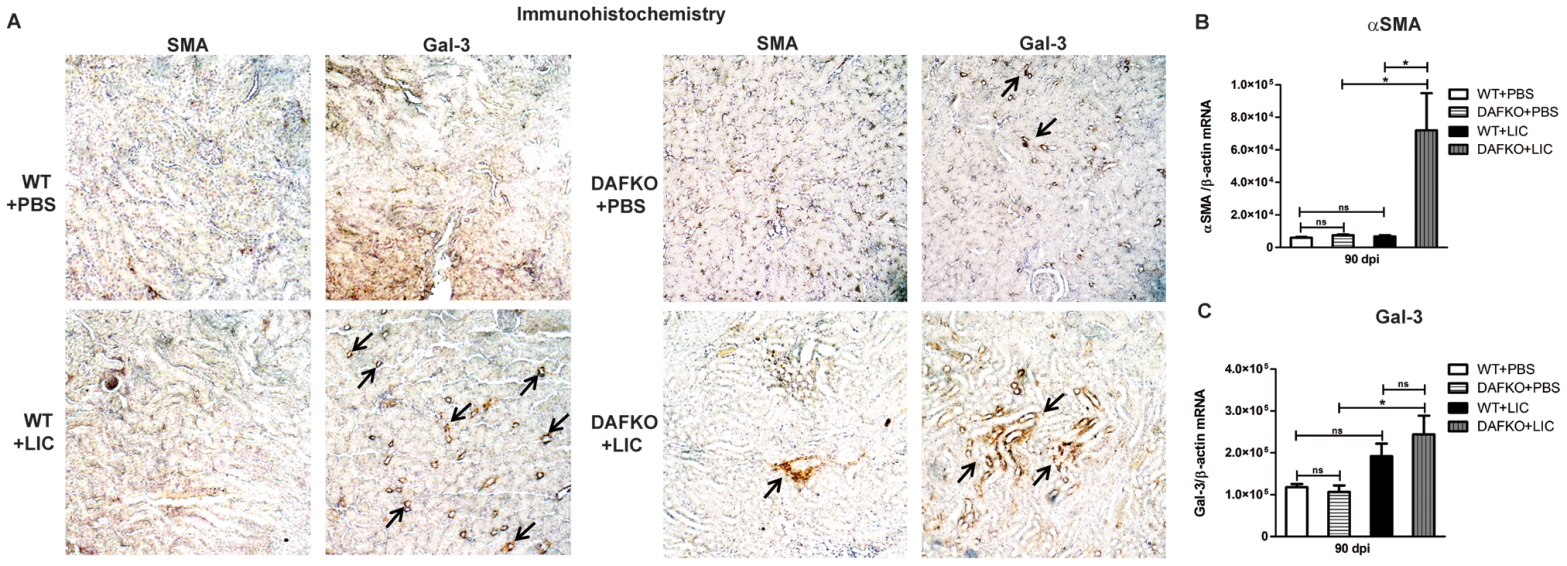
Chronic fibrosis in Daf1^−/−^ +LIC mice induces myofibroblast activation and enhanced galectin-3 expression. **A)** Immunohistochemistry of kidney sections from uninfected (WT+PBS and DAFKO+PBS) and LIC-infected (WT+LIC and DAFKO+LIC) mice with antiserum specific for α-smooth muscle actin (α-SMA) or galectin-3 (Gal-3) at 90 dpi (x200). Arrows indicate foci of antigen expression. Quantitative measurement of α-SMA (**B**) and Gal-3 (**C**) mRNA expression in kidney samples from uninfected (WT+PBS and DAFKO+PBS) and infected (WT+LIC and DAFKO+LIC) animals at 90 dpi. Bars represent the SEM of assays from a group of five to seven mice. Three pieces of each organ were analyzed in triplicate for q-PCR and normalized to host β-actin expression; *p<0.05, **^ns^** *p>*0.05.

Next, we investigated whether the observed chronic fibrosis was accompanied by myofibroblast activation and enhanced Gal-3 expression, as observed in models of renal fibrosis [29]. Enhanced α-SMA expression was observed only in Daf1^−/−^ infected mice (Figure 5 A). In contrast, both WT and Daf1^−/−^ infected mice showed enhanced Gal-3 expression in the renal tissue of infected mice when compared with the uninfected control group by IHC analysis (Figure 5 A). The analysis by qPCR showed significantly higher values only for Daf1^−/−^ infected mice (Figure 5 B–C, *p*<0.05).

### Cytokine levels in renal chronic fibrosis triggered by LIC infection

**Figure 6:**
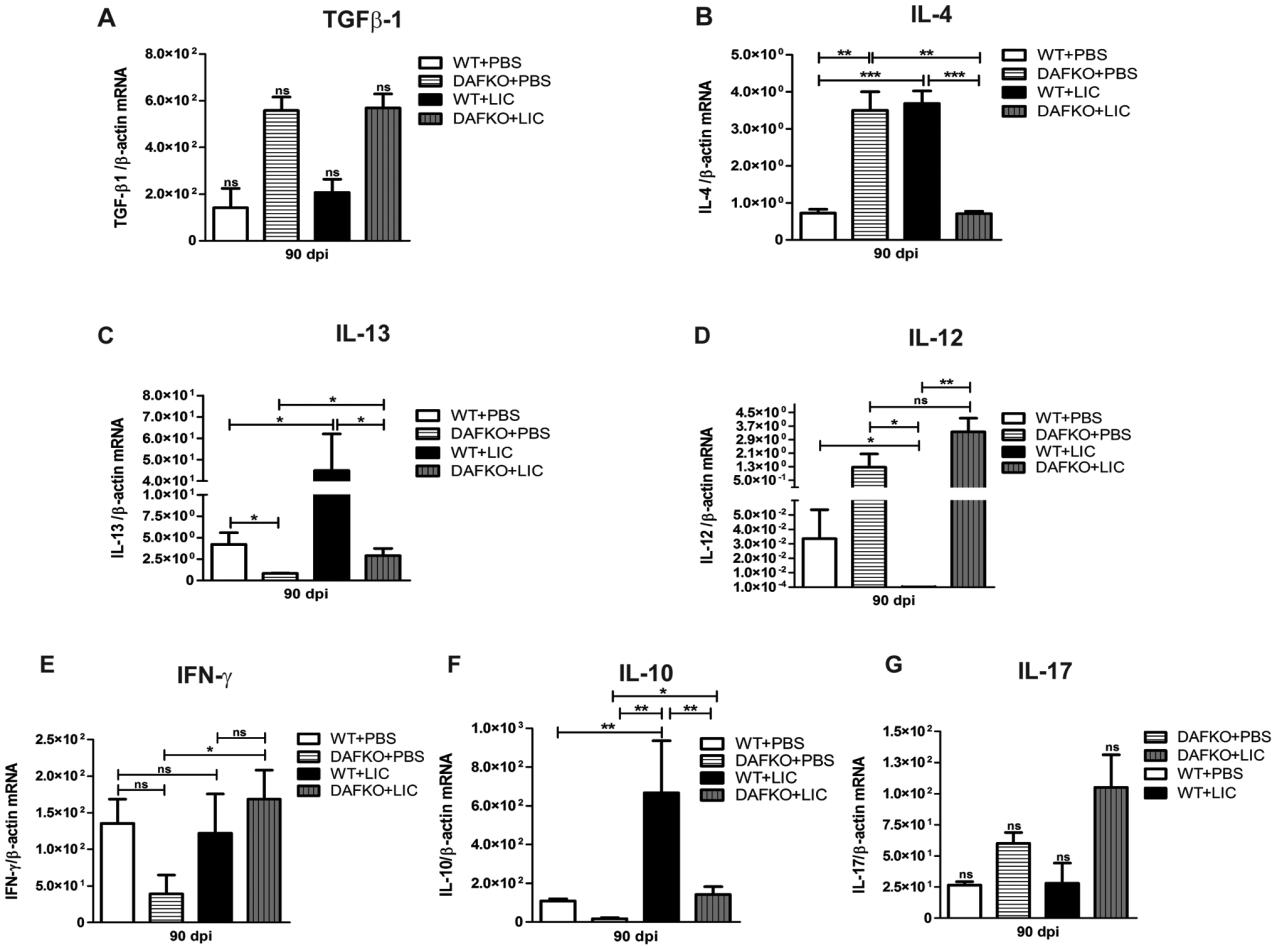
Cytokine levels in renal interstitial fibrosis triggered by LIC infection. Quantitative measurement of TGF-β1 (**A**), IL-4 (**B**), IL-13 (**C**), IL-12 (**D**), IFN-γ (**E**), IL-10 (**F**) and IL-17 (**G**) mRNA expression in kidney samples from uninfected (WT+PBS and DAFKO+PBS) or infected (WT+LIC and DAFKO+LIC) animals at 90 dpi. Bars represent the SEM of assays from a group of five to seven mice. Three pieces of each organ were analyzed in triplicate for q-PCR and normalized to host β-actin expression; *p<0.05, **p<0.01, ***p<0.001, **^ns^** *p>*0.05.

Previous studies have implicated TGF-β1 as an important mediator of renal fibrosis [2], [29]. However, TGF-β1 mRNA expression was similar in uninfected and infected mice at 90 dpi (Figure 6 A). Meanwhile, WT mice showed higher IL-4 mRNA expression than uninfected mice, while Daf1^−/−^ infected mice showed lower levels compared to Daf1^−/−^ uninfected animals (Figure 6 B, *p*<0.001 and *p*<0.01, respectively). IL-13 mRNA expression was up-regulated in both groups of infected mice (Figure 6 C, *p*<0.05). Significant down-regulation of IL-12 was observed in infected WT mice (*p*<0.05); meanwhile Daf1^−/−^ mice showed similar levels with and without infection (Figure 6 D). Significant up-regulation of IFN-γ was observed only in Daf1^−/−^+LIC animals (Figure 6 E, *p*<0.05). Finally, although elevated IL-10 mRNA levels were found in both WT and Daf1^−/−^ LIC-infected mice (Figure 6 F, *p*<0.01 and *p*<0.05, respectively), IL-17 expression was not different among the groups (Figure 6 G).

## Discussion

The kidney is the primary target of *Leptospira* during both subacute and chronic infection [5], [30]. In our model of LIC infection in C57BL/6J mice, we observed that all animals had mild to moderate renal inflammation at 14 dpi. However, it was not the typical interstitial nephritis described in guinea pigs, dogs or humans [30], and was only discrete to mild during the chronic phase in a small percentage of animals. This reduction in inflammation over time correlated with a significant decrease in the bacterial burden at 90 dpi and inversely correlated with levels of both IgM and IgG. Our results are relatively similar to those reported by others, where only some C57BL/6J mice had a score of 2 for inflammation at 28 dpi [31]. Interestingly, such restricted susceptibility was enough to trigger enhanced transcription of pro-collagen I at 90 dpi that correlated with a discrete degree of fibrosis present in some infected animals. Taken together, it was concluded that the C57BL/6J strain of mice is susceptible to LIC infection, which induces acute and chronic inflammation, and eventually translates into mild fibrosis correlated to some degree with the bacterial burden.

In order to explore the role of Daf1 in a LIC-induced murine infection, we studied the wild-type C57BL/6J strain of mice and the transgenic Daf1^−/−^ mice comparatively. The absence of Daf1 meant that LIC-infected mice showed higher mortality, as well as a greater kidney bacterial burden in the acute stage compared with WT animals. Moreover, whereas the kidney bacterial burden decreased at 90 dpi to barely detectable levels as in WT mice, the inflammation score rose. Interestingly, such differences correlated with a clearly higher level of fibrosis in the Daf1^−/−^ mice.

In the present work, only partial pathogenic mechanisms were unveiled. There was a significantly enhanced bacterial burden in the kidneys of Daf1^−/−^ mice during acute infection but a similar burden during the chronic stage. In comparison with WT, in previous studies, Daf1^−/−^ mice have shown higher acute viral load in murine CMV [32] but lower viral load in acute and chronic LCMV infection [33] or bacteremia after *Pneumococcal pneumonia* infection [34]. Reduced parasite load was also found in chronic *Trypanosome cruzi*-infected mice [35]. It is not clear why Daf1^−/−^ mice showed an early drop in survival and a higher bacterial burden at the start of the leptospiruric phase. It may partially be due to the reduced specific acute humoral response observed in Daf1^−/−^ mice since previous studies have demonstrated that this plays a major role [36]; however, our study does not exclude the possibility that the Daf1 molecule may play a more direct role, especially after the demonstrated cross-talk between complement, innate immune elements and inflammation [37], [38]. In this regard, a recent *ex vivo* study found that initial engagement of Daf1 by *E. coli* strains expressing Dr adhesins (and causing pyelonephritis) would allow their escape from phagolysosomal fusion, leading to non-destructive parasitism that allows bacteria to persist intracellularly [39]. The precise mechanism involved in our model will hopefully be clarified in future studies.

Previous studies have shown that outside membrane proteins (OMPs) from pathogenic leptospires are recognized by TLR2 of murine kidney tubular cells [40]. This recognition activates the transcription factor κB (NF-κB) and the mitogen-activated protein kinase (MAPK) pathway, triggering early inflammation and leukocyte recruitment [41] and later *in vitro* HeK-2 cell production of collagens through activation of the TGF-β1/Smad3 pathway [42]. However, by using several transgenic mice, a very recent study elegantly showed that TLR and NLR receptors as well as T lymphocytes are not required to generate *Leptospira*-induced renal fibrosis. Instead, the iNOS enzyme, known to play a role in *Leptospira*-induced interstitial nephritis [43], [44], was associated with the induction of renal fibrosis [45]. Given that in Daf1^−/−^ mice the increase in inflammation and fibrosis was not correlated with an increase in the bacterial burden present in the kidneys of chronically infected mice, we conclude that the major factors driving fibrosis in this model rely on the presence of leptospires during the early stages, and on the host response in the chronic phase without excluding a role for enhanced complement-mediated injury and/or reduced turnover of extracellular matrix components.

Gal-3 expression and infiltration of macrophages occurs early in LIC-induced nephritis and remains up-regulated, as shown in the UUO model [28]. However, enhanced α-SMA expression was observed only in the kidneys of infected Daf1^−/−^ mice. Both Gal-3 and α-SMA expression levels remain higher in the UUO model [28], continuously increasing macrophage recruitment, and probably justifying the clearly higher level of renal fibrosis observed in the UUO model by directly acting through TGF-β1-mediated myofibroblast activation and extracellular matrix production [46]. However, since it has been shown that Gal-3 forms lattices that promote cell-surface residence or the retention of cytokine and growth factor receptors, including receptors for TGF-β1, by interfering with their endocytosis, and that this retention of receptors leads to increased signaling [47], it should also be considered that enhanced levels of Gal-3 could modulate fibrosis indirectly. In addition, TGF-β1-independent mechanisms of renal fibrosis have also been reported in the UUO model [48]. Regarding IL-4, this cytokine it has been described in humans as a profibrotic cytokine promoting fibrocyte differentiation [2], whereas it has an inhibitory effect in mice [49] and therefore may explain the low levels at 90 dpi in Daf1^−/−^ mice. Hopefully, future studies will clarify if it has a role in WT mice. As expected, increased IL-13 levels were detected in both WT and Daf1^−/−^ infected mice at 90 dpi, since this cytokine has been reported to be a major profibrotic molecule that promotes fibrocyte differentiation [50]. The high levels of IFN-γ, IL-12 and IL-17 (although not significant) observed in the Daf1^−/−^ mice are probably a major contributor to the T-cell hyper-responsiveness of these mice [19], [22] and therefore may explain the similar or lower levels observed in the WT mice. IL-10 levels were elevated in both WT and Daf1^−/−^ infected mice. Interestingly, early enhanced IL-10 expression has been shown in other murine models of leptospirosis [51] and has been linked to death in humans [52] and hamsters [53] infected with leptospires.

**Figure 7:**
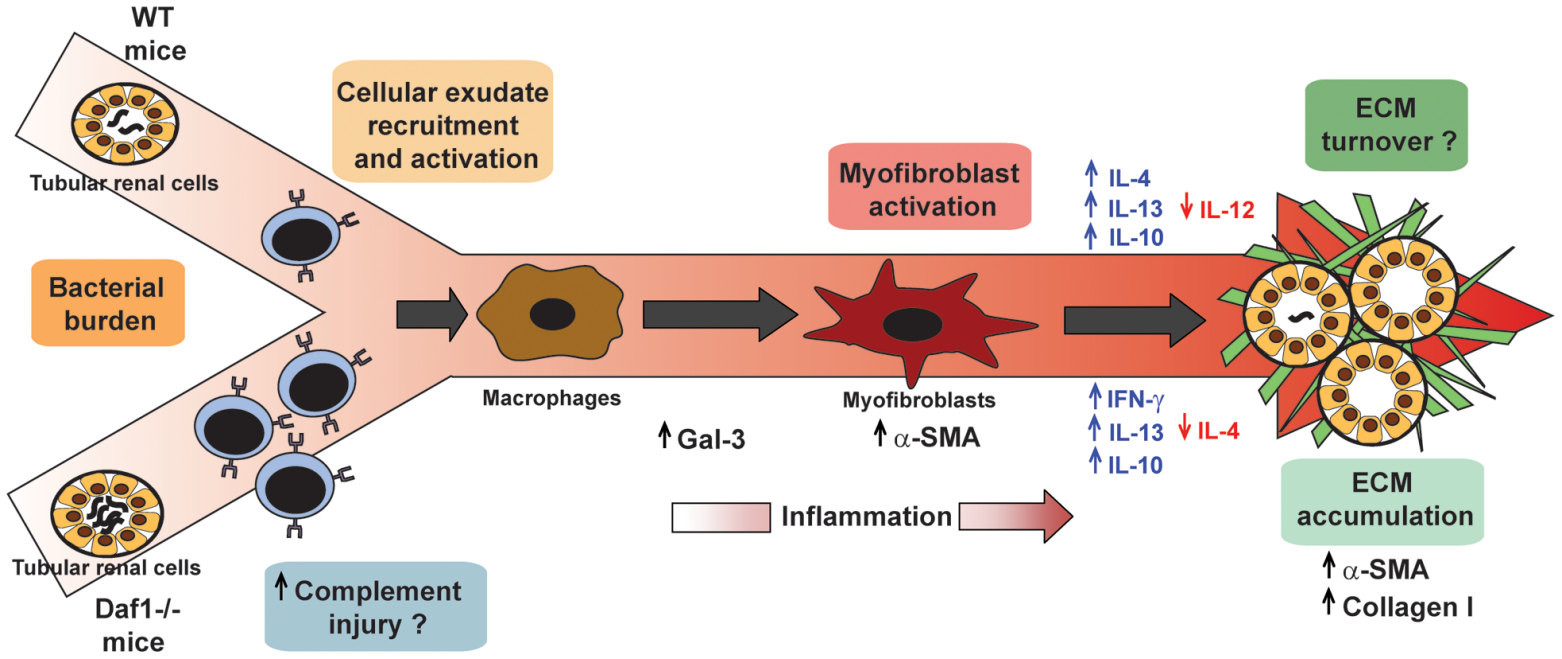
Model of leptospiral-induced murine chronic nephritis and renal fibrosis. Upon LIC colonization of proximal renal tubules of either C57BL/6J wild-type (WT) or Daf1^−/−^ mice, cellular exudate recruitment and activation occurs and precedes the arrival of macrophages, which after stimulation produce mediators such as galectin-3 (Gal-3) that activate quiescent fibroblasts and convert them into an α-SMA^+^ myofibroblast population. Myofibroblasts produce several molecules and orchestrate the production of ECM components and their extracellular assembly. In WT mice, down-regulation of IL-12 as well as up-regulation of IL-4, IL-10 and profibrotic cytokines such as IL-13 contributes to ECM accumulation. Of note, LIC-infected Daf1^−/−^ mice have a higher bacterial burden at the acute stage of infection, but barely detectable bacterial burden, increased IL-13, IL-10 and IFN-γ expression and decreased IL-4 expression during the chronic stages; these correlate with chronic inflammation, ECM deposition and renal fibrosis. Also, an alternative possibility is that the increased adaptive immune response against infection leads to the deposition of anti-leptospiral antibody followed by complement activation, which then contributes to kidney damage.

Based on the results of our experiments and very recent studies performed by others [45], it may be speculated that the presence of bacteria triggers the recruitment and activation of an early cellular exudate in the kidney [2]. It may be hypothesized that bacterial persistence contributes to non-resolving inflammation and a cellular exudate that sets the fibrogenic stage (priming) and triggers the activation and recruitment of extracellular matrix (ECM)-producing cells such as interstitial fibroblasts and circulating fibrocytes through Gal-3 and probably other molecules such as nitric oxide. Upon activation, ECM-producing cells assemble a multicomponent, integrin-associated protein complex that integrates input from various fibrogenic signals and orchestrates the production of ECM components and their extracellular assembly. Multiple cellular and molecular events, such as tubular atrophy and microvascular rarefaction, may promote scar formation and ensure a vicious progression to end-stage kidney failure [54] (Figure 7). Taken together, our results demonstrate that Daf1 plays a protective role in experimental leptospiral-induced fibrosis *in vivo*. Moreover, Daf1^−/−^+LIC animals constitute a suitable murine model for the study of leptospiral infections followed by the development of renal fibrosis.

It has been recently shown that asymptomatic renal colonization of leptospires in a region of high disease transmission is common, and may include people without serological or clinical evidence of recent infection with *Leptospira* persistence in the kidney [55]. However, the pathogenic significance of this finding, and particularly its connection with renal fibrosis, remains unknown [55]. Although scarce, some studies on human biopsies have shown an association between leptospirosis, interstitial nephritis and late-stage fibrosis [56]. In a recent case report of a young male patient, leptospirosis evolved to irreversible tubulointerstitial fibrosis that required chronic dialysis treatment [57]. In other studies of human biopsies, tubulointerstitial nephritis was associated indirectly with fibrosis based on increased collagen I, IV, TGF-β1 and Smad levels in the kidney, particularly when the disease was not treated with antibiotics [58]. In conclusion, there are no data showing to what degree *Leptospira* infection impacts on the incidence of human CKD, but the fact that it is a major human zoonosis, is frequently undiagnosed, and has a major impact on low social-economic populations, our and other [47] results associating even low bacterial persistence with renal fibrosis strongly suggest that it could be higher than expected, making *Leptospira*-triggered fibrosis a neglected event in a neglected disease. This should encourage more studies in order to clarify this issue.

## Materials and Methods

### Bacteria

The virulent *Leptospira interrogans* serovar Copenhageni (LIC) strain Fiocruz L1-130 and the culture conditions used have been described previously [59].

### Ethics Statement

All animal experiments were in compliance with the Argentine animal protection Law 14346 “*Malos tratos y actos de crueldad a los animales*”. The ethics committee of the “*Instituto de Biotecnología y Biología Molecular, CONICET-UNLP*”, in agreement with the International Guiding Principles for Biomedical Research Involving Animals (NIH, 1985), did not raise any concerns and approved our research protocol (identification number 001/12). All animals received water and food *ad libitum*. All efforts were made to minimize suffering.

### Animals and experimental design

C57BL/6J wild-type or C57BL/6J Daf1^−/−^ mice, aged 3–4 weeks, were injected intraperitoneally (ip) with 0.2 ml of PBS (uninfected control group) or 0.2 ml of PBS containing 10^6^ LIC strain Fiocruz L1-130. Mice were monitored daily and euthanized by CO_2_ overdose at 14 and 90 dpi (5–7 mice were used for each time point) and their blood and kidneys were then harvested. Routinely, one part was frozen at −80°C for further studies and the other was fixed with buffered 4% paraformaldehyde for histological examination and immunoperoxidase labeling. Mortality of C57BL/6J Daf1^−/−^ LIC-infected mice at 3 or 4 dpi was unexpected and presented no previous signs.

### Histopathology and immunohistochemistry

Nephritis was graded blindly by a pathologist on a scale of 0–4 in a whole longitudinal section of the organ following previously published criteria for scoring kidney injury present in leptospirosis [31]. The PS technique was carried out as previously described [60], [61], [62]. Digital image analysis was used to quantify the amount of red-stained collagen fibers as previously described [28] using a Nikon E200 microscope with a Tucsen TCC 5.0 digital camera and the software provided by the manufacturer. The IHC procedure have been previously described [43] using anti α-SMA, (Clone 1A4, Dako), Gal-3 (Clone M3/38), MAC (Abcam 55811) and an anti LipL32 (a gift from Dr Nascimento, Butantan Institute). Acute necrotizing pancreatitis was used as a positive control for the MAC IHC [63], [64].

### DNA-RNA isolation and RT-PCR

Total DNA or RNA was isolated from the kidney by mechanical homogenization and Trizol (Invitrogen), as recommended by the manufacturer. The DNA or RNA was quantified with a Nanodrop spectrophotometer ND-1000. Prior to cDNA synthesis, DNase treatment was performed with an RNasefree DNase Kit (Qiagen). cDNA was synthesized from 500 ng of total RNA with 15 mM of random hexamers and MMLV reverse transcriptase (Promega), according to the manufacturer’s instructions.

### Real-time PCR

The q-PCR studies were performed with a Line-Gene K instrument and software (Bioer). The 5x HOT FIREPol EvaGreenqPCR Mix Plus was used for all reactions, following the manufacturer’s instructions. Standard cDNA samples with 10-fold serial dilutions were used for PCR efficiency calculations. Cycle threshold (Ct) values were obtained for each individual reaction, and the Ct of the host-expressed β-actin was subtracted to obtain pro-collagen type I, SMA, Gal-3, TGF-β1, IL-4, IL-10, IL-12, IL-13, IL-17 and IFN-γ normalized values, respectively [35], [43]. To obtain bacterial burden values, 16SDNA bacterial gene was amplified and the number of bacteria was referred to that of the host cells [35]. The primer sequences and sizes of the amplified fragments are shown in Table 1.

**Table 1:** Primers used in q-PCR assays.

Gene product	Primer	Primer sequence (5′-3′)	Amplicon length (bp)
16S	F	CATTCATGTTTCGAATCATTTCAAA	331
	R	GAAACACGGACACCCAAAGTA	
Pro-collagen type I	F	TTCACCTACAGCACCCTTGTG	66
	R	GATGACTGTCTTGCCCCAAGTT	
α-SMA	F	GCTCTGCCTCTAGCACACAA	150
	R	GCCAGGGCTACAAGTTAAGG	
Gal-3	F	GACCACTGACGGTGCCCTAT	149
	R	GGGGTTAAAGTGGAAGGCAA	
TGF-β1	F	TGCGCTTGCAGAGATTAAAA	82
	R	AGGTAACGCCAGGAATTGTTGCTA	
IFN-γ	F	CTTGGATATCTGGAGGAACTGGC	234
	R	GCGCTGGACCTGTGGGTTGTTGA	
IL-4	F	CATCGGCATTTTGAACGAGGTCA	240
	R	CTTATCGATGAATCCAGGCATCG	
IL-10	F	CCAGTTTTACCTGGTAGAAGTGATG	324
	R	TGTCTAGGTCCTGGAGTCCAGCAGACTCAA	
IL-12	F	ATGGCCATGTGGGAGCTGGAGAAAG	225
	R	GTGGAGCAGCAGATGTGAGTGGCT	
IL-13	F	GACCAGACTCCCCTGTGCAA	121
	R	TGGGTCCTGTAGATGGCATTG	
IL-17	F	ACCGCAATGAAGACCCTGAT	83
	R	TCCCTCCGCATTGACACA	
β-actin (DNA)	F	GGCTGTATTCCCCTCCATCG	241
	R	CCAGTTGGTAACAATGCCATGT	
β-actin (cDNA)	F	CGTCATCCATGGCGAACTG	60
	R	GCTTCTTTGCAGCTCCTTCGT	

### ELISA

Detection of total IgM and IgG-specific antibodies against *Leptospira* was performed as described previously [43].

### Statistical analysis

Data were expressed as the mean+S.E.M. and were analyzed by one-way analysis of variance (ANOVA) followed by Bonferroni multiple comparison test to determine significant differences between groups. *p* values <0.05 were considered statistically significant.
